# The probabilistic backbone of data-driven complex networks: an example in climate

**DOI:** 10.1038/s41598-020-67970-y

**Published:** 2020-07-13

**Authors:** Catharina E. Graafland, José M. Gutiérrez, Juan M. López, Diego Pazó, Miguel A. Rodríguez

**Affiliations:** Instituto de Física de Cantabria, CSIC–Universidad de Cantabria, Avenida de Los Castros, 39005 Santander, Spain

**Keywords:** Climate sciences, Complex networks, Statistics

## Abstract

Complex systems often exhibit long-range correlations so that typical observables show statistical dependence across long distances. These teleconnections have a tremendous impact on the dynamics as they provide channels for information transport across the system and are particularly relevant in forecasting, control, and data-driven modeling of complex systems. These statistical interrelations among the very many degrees of freedom are usually represented by the so-called correlation network, constructed by establishing links between variables (nodes) with pairwise correlations above a given threshold. Here, with the climate system as an example, we revisit correlation networks from a probabilistic perspective and show that they unavoidably include much redundant information, resulting in overfitted probabilistic (Gaussian) models. As an alternative, we propose here the use of more sophisticated probabilistic Bayesian networks, developed by the machine learning community, as a data-driven modeling and prediction tool. Bayesian networks are built from data including only the (pairwise and conditional) dependencies among the variables needed to explain the data (i.e., maximizing the likelihood of the underlying probabilistic Gaussian model). This results in much simpler, sparser, non-redundant, networks still encoding the complex structure of the dataset as revealed by standard complex measures. Moreover, the networks are capable to generalize to new data and constitute a truly *probabilistic backbone* of the system. When applied to climate data, it is shown that Bayesian networks faithfully reveal the various long-range teleconnections relevant in the dataset, in particular those emerging in El Niño periods.

## Introduction

Due to the widespread interest of the scientific community in data science, an increasing body of research in the field of complex networks is now focusing on the development of graph machine learning algorithms for analysis and prediction^1^. Some relevant applications include link prediction^2,3^, network embedding^4^, pattern mining^5^, and graph neural networks^6^. Most of this research is currently oriented towards the application of deep learning methods^7–10^. However, there are a number of traditional machine learning methods that could be used in the modern context of complex graph analytics and prediction. For instance, Bayesian network (BN) models^11^ are a sound and popular machine learning technique used to build tractable probabilistic models from data using auxiliary graphs—representing the most relevant (pairwise and conditional) dependencies among the variables needed to explain the data as a whole (maximizing the likelihood of the underlyingGaussian model). These models have been successfully applied in a few particular climate applications, such as probabilistic weather prediction^12^ or causal teleconnection analysis^13,14^.

In recent years, the most popular approach to modeling and obtain the patterns of teleconnections in complex systems, like for instance climate, is based on correlation networks (CNs)^15–22^. The difference between CNs and BNs is that, whereas the former are exclusively built considering pairwise dependencies (e.g., correlations) between variables (based on the choice of an arbitrary threshold), the latter use more sophisticated learning methods to model also conditional dependencies, i.e., dependencies between two (sets of) variables, given a third (set). Here we show that this results in sparser, non-redundant, networks with a complex topology. We find that the topology of an optimal BN consists of a community structure with maximal entropy at all hierarchical levels. Moreover, as we shall show here, from a probabilistic perspective CNs and BNs lead to very different empirical Gaussian models. The resulting CN distribution function is either too simple (mostly dominated by local information, therefore, unable to predict teleconnection patterns for high correlation threshold values) or too noisy (containing too many parameters for small thresholds) and prone to overfitting. In contrast, the BN distribution function is a parsimonious representation suitable for analysis and probabilistic inference.

**Figure 1:**
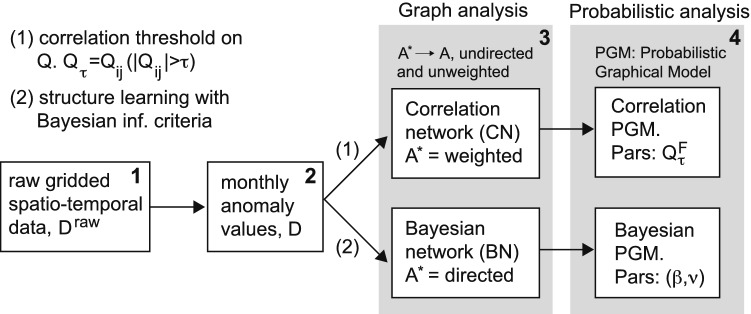
Schematic illustration of construction of Correlation Networks (CNs) and Bayesian Networks (BNs) and their associated Probabilistic Graphical Models (PGMs). Gray boxes indicate the type of analysis performed on either the networks or the PGMs. "Climate network construction" section describes the process from raw spatio-temporal data to monthly anomaly values, box 1 to 2. "Determining CN structure from data" and "Learning BN structure from data" sections describe the network construction process between boxes 2 and 3. "Probabilistic Gaussian graphical models" and "Probabilistic CN models" and "Probabilistic BN models" sections describe the process between boxes 3 and 4.

Summarizing, we shall advocate here the use of BNs as non-redundant graph representations of complex data, suitable for probabilistic modeling and analysis with complex network measures. For the case of climate data^23^, we shed light on the construction of the proposed BNs and compare with the usual CNs approach by characterizing the topology of both type of networks. This graph theoretic analysis shows why CN topologies are inherently redundant, while BNs are not. Moreover, by using machine learning techniques, we analyse the networks as Probabilistic Graphical Models (PGMs) that have objective information content to soundly present the underlying Gaussian model. It will become clear that redundancy in topology is associated with the surge of meagre model parameters, so that this redundancy hinders the use of CNs to extrapolate meaningful features in the case of new data. We illustrate this with a particular extrapolation study in which we query the networks to predict the teleconnections that appear during a climatic event (El Niño). A schematic overwiew of the network construction and analysis in this work is given in Fig. 1.

## Results

### Climate network construction

The construction of CNs and BNs in climate (boxes 1, 2 and 3 in Fig. 1) is illustrated using a global temperature dataset previously used in many studies^16,17,21^ that shows well-known properties characterized by the interplay of strong local and weak long-distant (teleconnections) dependencies. In particular, we use monthly surface temperature values on a global $10^\circ$ resolution (approx. 1000 km) regular grid for a representative climatic period (1981 to 2010), as provided by the ERA interim reanalysis dataset^24^. The temperature anomaly values of an arbitrary month, (i.e., the local differences with respect to the mean temperature), $X_i$ at grid point *i* are the variables of interest and represent the ($18 \times 36 = 648$) nodes of the network models. The anomaly samples $\varvec{{\mathscr{D}}}$ (that do not distinguish between type of months) are obtained subtracting the sample mean over all years: removing the annual cycle (the 30-year mean values, month by month) from the raw data $\varvec{{\mathscr{D}}}^{\text{raw}}$—For a particular grid point *i* the *k*th realization $d_i^k$ in the sample that is associated with, for instance, the month $k =$ Jan:1985 is obtained from the raw data value $(d_{i}^{\text{Jan:1985}})^{\text{raw}}$ by $d_i^k = (d_{i}^{\text{Jan:1985}})^{\text{raw}} - \sum _{y=1}^{30}(d_{i}^{\text{Jan:}y})^{\text{raw}}/30$. The same procedure is followed to obtain the anomaly value for any month and year in the time series (at any particular grid point).

The network size (number of edges) and topology of these connections determine the complexity and properties of the probabilistic models constructed from the dataset and have implications for both model interpretation and ability of generalization to new data. Hence, we shall discuss the different results in this paper in light of the network size.

**Figure 2:**
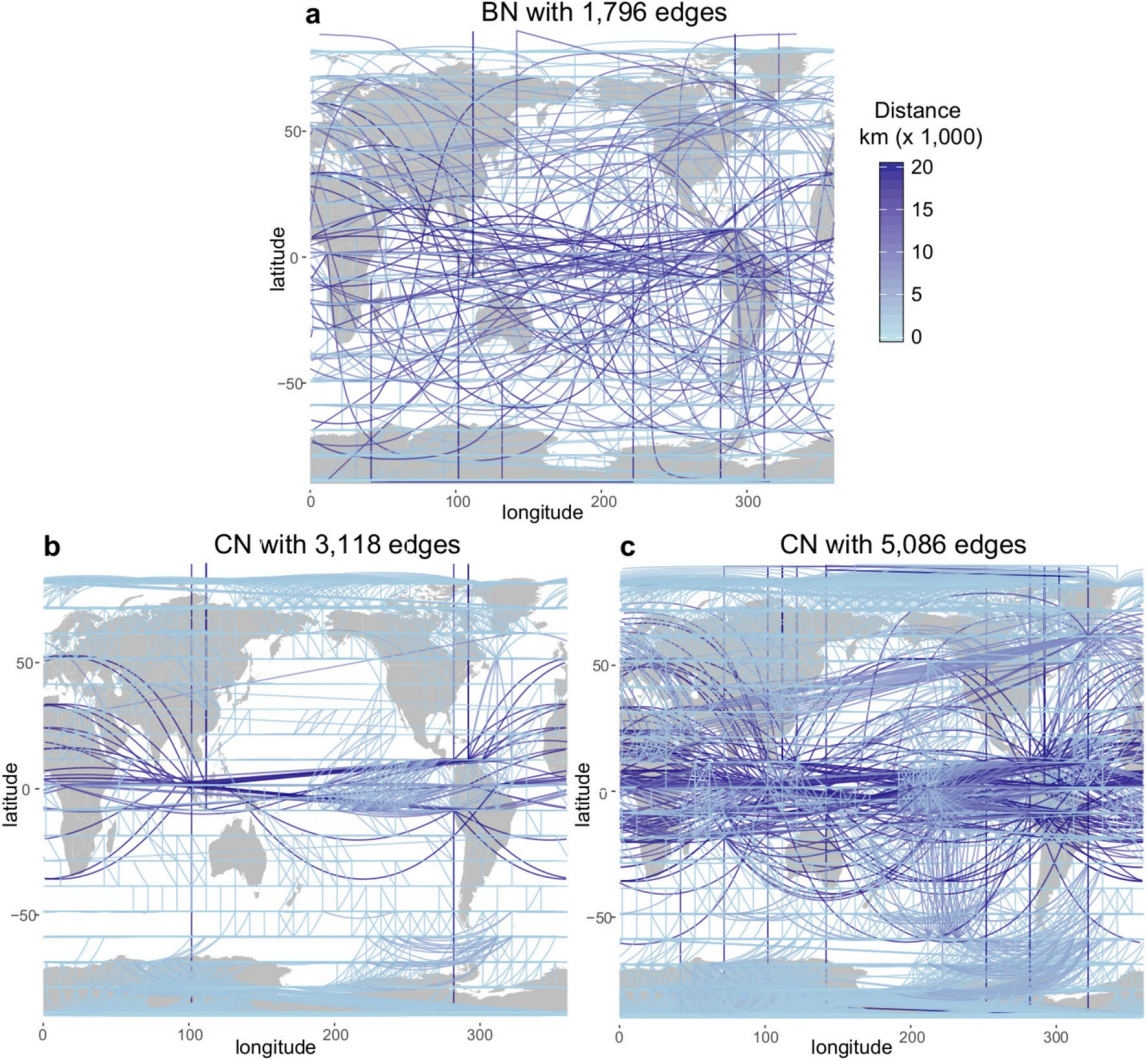
(**a**) Bayesian Network (BN) with 1,796 edges and Correlation Networks (CNs) consisting of (**b**) 3,118 edges and (**c**) 5,086 edges. The networks are constructed from monthly surface temperature values on a global $10^\circ$ resolution (approx. 1000 km) regular grid for the period 1981 to 2010. The network represents the dependencies between temperature anomaly values in the gridboxes. Edges are coloured as a function of the distance between the gridpoints they connect. Maps in this Figure were created using the R-packages maps v3.2.0^26^, geosphere v1.5-7^27^ and ggplot2 v3.1.0^28^ that respectively provided the world-map and tools for distance calculation and visualization (https://ggplot2.tidyverse.org/).

The construction of CNs is somehow arbitrary since it is controlled by the choice of the threshold $\tau$ on the sample correlation matrix $\varvec{Q}$, above which variables are considered to be *connected* in the resulting graph. A number of studies have analysed the effects of different thresholds in the resulting topological properties of the network^25^. It was found that different features of the system are revealed at different threshold levels^17^ and, as a consequence, the choice of the threshold has to reflect a trade-off between the statistical significance of connections and the richness of network structures unveiled. Small correlation thresholds are needed to capture the ‘weak’ teleconnections in the case of climate networks^16^, but this inevitably leads to a high degree of spurious over-representation of the local (strongly correlated) structures, i.e., redundancy. For example, Fig. 2b, c show two different CNs obtained from the temperature data considering two different illustrative thresholds $\tau =0.50$ and $\tau = 0.41$, respectively, that yield networks of 3,118 and 5,086 links. On the one hand, the $\tau =0.50$ CN in Fig. 2b shows very highly connected local regions (e.g., the tropics and Antarctica), and only a few long-distance links corresponding to teleconnections. On the other hand, the $\tau = 0.41$ CN in Fig. 2c shows a high density of both local and distant links, therefore, a high degree of redundancy for characterizing the main physical features. In reality, it is difficult to find the *appropriate* threshold, or any objective criteria to select it, to obtain a network that is able to represent the main features underlying the data without arbitrariness.

In contrast, BNs are built from data using a machine learning algorithm which encodes in a network structure the (marginal and conditional) dependencies among the variables that allow to best explain the data in probabilistic terms. In this case, the network has a corresponding probabilistic model (a Gaussian distribution in this example), given by a network-encoded factorization which implies the same underlying dependency structure (see "Probabilistic BN models" section). Learning proceeds iteratively, including new edges (dependencies) at each step, so that one maximizes the model likelihood, while penalizing complexity (see "Learning BNs" section). For instance, Fig. 2a shows a BN learnt from the temperature data with only half of the links as the CN in Fig. 2b. In contrast to both CNs shown, the BN is able to capture both local and long-distant structures without redundancy, exhibiting a good balance between local and long distance links.

### Community structure

**Figure 3:**
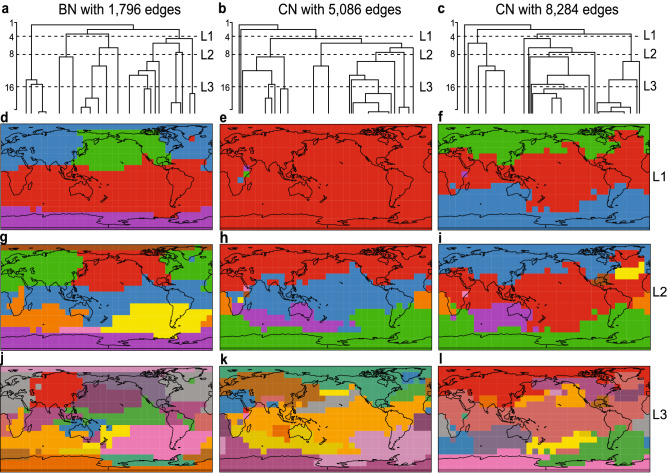
Dendrograms (first row) and community division (2nd to 4th row) of BN with 1,796 edges (first column) and CNs with 5,086 (second column) and 8,284 (third column) edges as found by the edge-betweenness-community detection algorithm. The vertical branches represent communities, which branch off as the algorithm proceeds. The horizontal distance between the two branches adjacent to a given branch is an upper bound of the size of that community. The height of levels L1, L2 and L3 in the dendrograms indicates the number of communities in which the networks are divided in the 2nd, 3rd and 4th row, respectively. Instead of all 648 (number of vertices) levels (divisions of the network by the algorithm) only the first 20 levels are represented in the dendrograms. Maps in this Figure were created using the R-package visualizer v1.5.1 that forms part of the climate4r open framework (http://www.meteo.unican.es/climate4R)^29^.

We deepen the investigation of the topology of BNs and CNs of different sizes using well-established complex network tools (box 3 in Fig. 1). The topological analysis is done on unweighted and undirected networks of dependencies, obtained in the case of CNs by converting the thresholded covariance matrix to binary format, and in the case of BNs by converting the Directed Acyclic Graphs (DAGs) of dependencies to an undirected graph. Results on a selection of complex measures that characterize the global and local structure of the networks are shown in the Supplementary information. Here we focus on the distinctively different community structure of BNs and CNs. We analysed the partition of our networks in betweenness-based communities. In climate sciences (node or edge) betweenness is an important proxy that is used to characterize climate topology^16,17,30^. The betweenness aims to reveal the extent to which edges or nodes are a key for efficient (shortest paths) interconnection over distant places on the network. Results on a climate data adapted betweenness measure to our BNs and CNs are found in Supplementary information, Figure S1.

Betweenness-based climate communities are visually easy to interpret; vertices in the same climate community communicate whatever deviation of their mean temperature, and the community search algorithm iteratively divides the network in a different number of communities allowing the user to visualize different scales or levels in the network topology that capture different physical features of a network. The concepts of communities and betweenness are related. Edges that lie between communities can be expected to have high value of edge-betweenness (see "Betweenness centrality" and "Community detection" sections), as such, iterative removal of edges with high betweenness consistently splits a network in communities; this technique is used in the community search algorithm^31, 32^ that we used to partition our networks in edge-betweenness communities.

Figure 3 shows results on communities for a BN with 1,796 edges and two CNs with 5,086 ($\tau = 0.41$) and 8,284 ($\tau = 0.33$) edges at three different levels of community partition. The BN shows, already at the first partition level, Fig. 3d, a high connectivity among variables in the tropics, the poles and north pacific ocean are highlighted. At the second level, Fig. 3g, the BN exhibits teleconnections among north and mid Atlantic, east and west Pacific, and Indian oceans. Communities continue to split as one goes on removing edges with the highest betweenness. At the third level, Fig. 3j, some of the existing communities consist of spatially separated clusters that are linked through long-range edges, emphasizing the existence of teleconnections and its important role in the community structure of BNs.

In contrast, the community partition of the CN of size 5,086 is less informative at the first level, Fig. 3e. The whole globe is fully connected with the exception of three separated gridboxes. These three communities correspond to three isolated variables in the topology to which the algorithm was forced to assign three uninformative communities. In general, the communities arising from CNs contain less significant information as compared with BNs at the same level of community partition. This is due to the poor performance of CNs in the job of connecting long-distant variables (see Supplementary information Figure S2 and Figure S3, in which the size of the largest connected component is visualized with respect to the number of edges in the network). As such, for all CNs that contain less than 5,086 edges a similar first level is obtained. At the second level, Fig. 3h, using more communities, the CN partition captures the connectivity of the tropics, that already appears in the BN at the first level, Fig. 3d. Thereby, some of the separate communities also found at the second BN level, Fig. 3g, get highlighted. Note that the teleconnection of the tropics with the north Atlantic Ocean is not seen at this early stage of the partition in communities for the CN. At the third level, Fig. 3k, many small climate communities pop up in the CN but the presence of very many redundant links in the giant tropical component, which are also clearly apparent in Fig. 2c, hinder the algorithm from an efficient partition of the giant component. At the third level of the community partitioning of a CN with more edges, Fig. 3l (8,284 edges), the giant tropical community of the CN still remains unbroken. At a deeper level (not shown) the giant teleconnected component will be broken by the algorithm after proliferation of many communities with little information content.

The dendrograms in Fig. 3a–c serve as overviews of the community partition process for the three networks discussed above. A significant difference in the community fragmentation is apparent: While CNs undergo a strongly inhomogeneous division in communities, the BN partitions in a highly uniform fashion.

**Figure 4:**
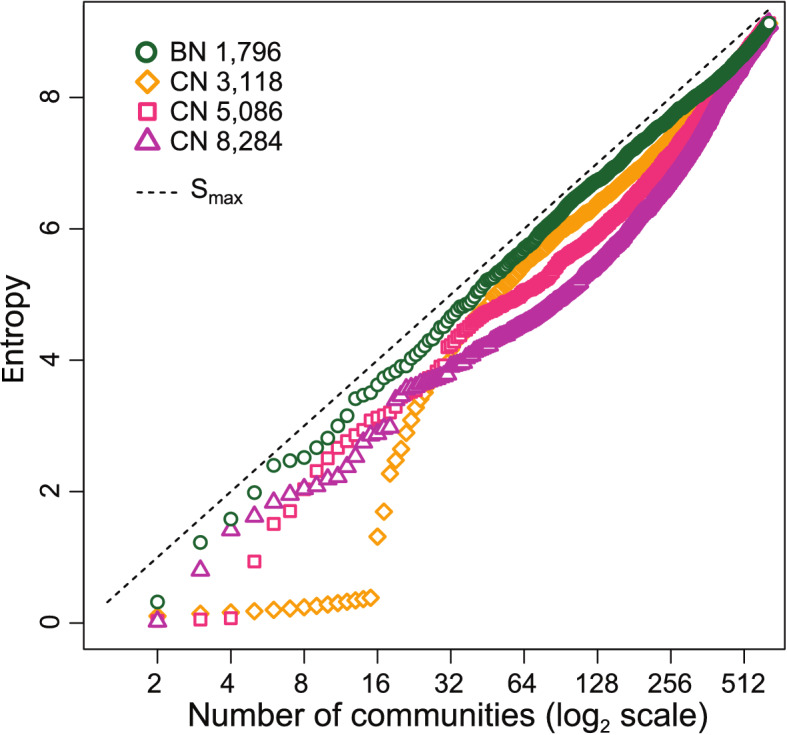
Entropy $S = - \sum _{\alpha =1}^{N_c}\omega _\alpha \log _2\omega _\alpha$ versus number of disjoint communities $N_c$ in which the network is partitioned. Results are displayed for a BN of 1,796 edges (green) and for CNs of respectively 3,118, 5,086 and 8,284 edges (orange–magenta). The dashed line represents the maximum entropy $S_{\text {max}}$ that can be obtained for the corresponding number of disjoint communities $N_c$.

These observations can be made quantitative by calculating the *entropy*, *S*, of the community partition for each type of network (see "Entropy" section). Suppose that we have our network partitioned in a number, $N_c$, of disjoint communities and we ask ourselves what is the information content of this community structure. In other words, how much information gain (on average) we would obtain by determining that a random node belongs to a certain community. If the entropy is high, this means that every time we ascertain that a site belongs to a given community we gain much information on the structure. Conversely, if entropy is low the average information gain we obtain by this process is small, on average. The maximum entropy corresponds to an even distribution of the sites among existing communities, while a low entropy would mean that some communities are much larger than others, and so, there is a much higher probability that a site, picked at random, belongs to the most populated communities. The amount of information conveyed in this case by specifying the community structure is lower. One can prove (see "Entropy" section) that the maximum entropy corresponds to $S_{{\mathrm{max}}} = \log _2 N_c$, where $N_c$ is the number of communities. In Fig. 4 we plot *S* as a function of the number of communities for the optimal BN and for CNs of different sizes. One can see that *S* for the optimal BN of 1,796 edges, corresponding to the BN in Fig. 2a, attains values close to the maximal entropy for *any* number of communities $N_c$, from early stages in the community splitting process (where only 4 to 6 communities are present) to later stages (when a few hundred communities have been found). In contrast, the entropy of CNs (no matter the threshold chosen) is always below the BN optimal case. This clearly shows that the community structure of small, sparse BNs have much larger amounts of information content than their CN counterparts.

In addition to the betweenness-based-algorithm, we used other community detection algorithms based on other criteria such as maximal modularity (Louvain^33^), shortest description length of a random walker (Infomap^34^), or the probability of occurrence of a community in a random network (OSLOM^35^). However, these algorithms do not allow to investigate community structure by levels of depth, as they only return one (to three—in the case of OSLOM—) optimal community partition(s), with a number of communities that depends on the specific criterion/algorithm. For almost all partitions that we obtained by these algorithms there exist an equivalent partition of the betweenness-based-algorithm. The results of other community detection algorithms thus, somehow, substantiate the partitions that emerge from the betweenness-based-algorithm, but the latter is preferred for our study as it provides community structure at different depth levels.

### Probabilistic model construction and cross-validation

**Figure 5:**
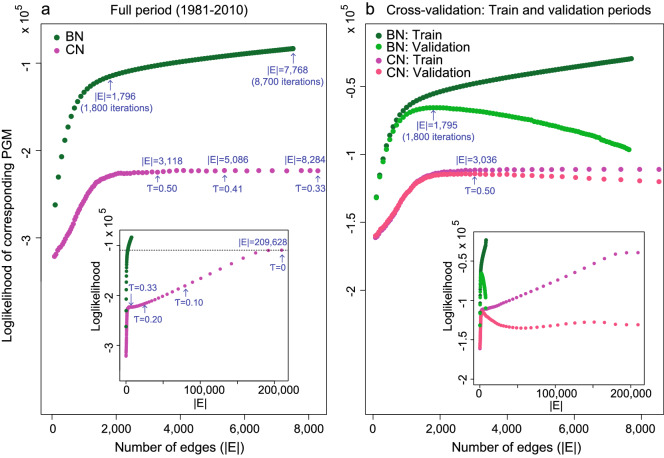
(**a**) Log-likelihood values $\log {\mathrm{P}}({\varvec{\mathscr{D}}}_{{\mathrm{c}}}{{|}}\mathrm{PGM}_{{\mathrm{c}})}$ versus number of edges (|*E*|) of Bayesian (green) and correlation (magenta) PGMs that are learnt from the complete dataset ${\varvec{\mathscr{D}}}_{{\mathrm{c}}}$ and (**b**) Log-likelihood values $\log {\mathrm {P}}({\varvec{\mathscr{D}}}_{{\mathrm{t}}}{{|}}\mathrm{PGM}_{{\mathrm{t}}})$ (green and magenta) and $\log {\mathrm {P}}({\varvec{\mathscr{D}}}_{{\mathrm{v}}}{{|}}\mathrm{PGM}_{{\mathrm{t}}})$ (light green and light magenta) versus number of edges of Bayesian (green and light green) and correlation (magenta and light magenta) PGMs that are learnt with the train dataset ${\varvec{\mathscr{D}}}_{\mathrm{t}}$. In (**a**) and (**b**) outer windows are a zoom of the small windows inside. Some log-likelihood values of correlation PGMs are labelled with the threshold $\tau$ which was used to construct the CN and some log-likelihood values of Bayesian PGMs are labelled with the number of iterations that was used by the structure learning algorithm to construct the BN. In (**b**) these labels are placed by the BN and CN for which the corresponding PGM obtains a maximum value of $\log {\mathrm {P}}({\varvec{\mathscr{D}}}_{{\mathrm{v}}}{{|}}\mathrm{PGM}_{{\mathrm{t}}})$. In the small window of (**a**) the dotted line represents the log-likelihood value of a complete Correlation PGM of size 209,628, corresponding to $\tau = 0$.

Here we analyse from a probabilistic perspective the networks built in the previous sections by extending the graphs to full Probabilistic Graphical Models (PGMs), in which edges on the graph will represent parameters in the probability density function (box 4 in Fig. 1). In this paper we work with the natural choice of multivariate Gaussian distributions as PGMs. On the one hand, the probability density function for a CN is constructed by specifying the covariance matrix elements from the empirical correlations $Q_{ij}$ that are above the fixed threshold (i.e., for those edges that are present in the CN graph). On the other hand, the probability distribution function for a BN is represented by a factorization of conditional multivariate Gaussian probabilities and the parameters are the linear regression coefficients of a variable on its conditioning variables (i.e., those that are connected by an edge in the graph with a parent–child relation). See sections "Probabilistic Gaussian graphical models" and "Probabilistic CN (BN) models" for more information on the extension of CNs and BNs into PGMs and their particular encoding of the multivariate Gaussian density function.

A key problem in machine learning is whether the models learnt from a training data sample can capture general and robust features of the underlying problem, thus providing out-of-sample extrapolation capabilities. This property is known as *generalization* and it is typically assessed in practice using a test data sample (or, more generally, by cross-validation) to check whether the model is *overfitted* (the model explains very well the training data but fails to explain the test).

Once networks are extended to PGMs one can compare them using the log-likelihood $\log {\mathrm {P}}({{\varvec{{\mathscr{D}}}}}|\mathrm{PGM})$, where ${\varvec{\mathscr{D}}}$ is the dataset. The log-likelihood can be interpreted as the probability of a given dataset ${\varvec{{\mathscr{D}}}}$ when ${\text {P}}$ is modelled by a certain PGM (see "Log-likelihood definition and calculation" section, for details). The log-likelihood compares models that encode the same type of density function ${\text {P}}$, but with different parameters, and should be interpreted comparatively; the log-likelihood value of model A is not very meaningful in absolute terms, however, if log-likelihood of model A is higher than that of model B, one can conclude that model A explains the data better than model B. In this work all types of PGMs and, along them, all networks of different sizes encode a multivariate Gaussian distribution over a constant dimensional variable space, making the log-likelihood an adequate comparative measure^36^.

First, we use the log-likelihood to compare the fit of CNs and BNs to the complete dataset ${{\varvec{\mathscr{D}}}}_{{\mathrm{c}}}$ by calculating ${\mathrm{P}}({{\varvec{{\mathscr{D}}}}}_{{\mathrm{c}}}|{\mathrm{PGM}_{\mathrm{c}}})$ for networks of various sizes, in which ${\mathrm{PGM}}_{{\mathrm{c}}}$ refers to the PGM that is learnt from the complete dataset ${{\varvec{\mathscr{D}}}}_{{\mathrm{c}}}$. Next, we use the log-likelihood to assess the *generalization* capability of the models, calculating cross-validated log-likelihood values ${\mathrm {P}}({{\varvec{\mathscr{D}}}}_{{\mathrm{v}}}|{{\mathrm{PGM}}_{{\mathrm{t}}}})$, obtained by splitting the data into two halves, one for training ${\varvec{\mathscr{D}}}_{{\mathrm{t}}}$ and one for validation ${{\varvec{\mathscr{D}}}}_{{\mathrm{v}}}$, where ${{\mathrm{PGM}}_{{\mathrm{t}}}}$ denotes the PGM that is learnt from from the training dataset ${{\varvec{\mathscr{D}}}}_{{\mathrm{t}}}$. Figure 5a and 5b show the results for complete-dataset-fit and generalization, respectively.

Figure 5a shows ${\mathrm{P}}({\varvec{\mathscr{D}}}_{{\mathrm{c}}}|{\mathrm{PGM}}_{{\mathrm{c}}})$ as a function of the network size for CNs and BNs. Addition of parameters to a model facilitates it to explain the data on which it was trained and, thus, this should increase log-likelihood. Figure 5a shows that the amount of gain in log-likelihood depends on the type of model; adding parameters (edges) to the BN turns out to be very efficient, yielding a gain in log-likelihood. However, when adding parameters to the CN it becomes efficient only up to a certain size (around $2\times 10^3$ edges). Once this size is exceeded the log-likelihood only continues to grow after a great amount of parameters have been added (indeed, the growth continues around $3\times 10^4$ edges, see inset of Fig. 5a). The figure shows that CNs and BNs of similar size strongly differ in the amount of data their associated PGMs explain, BNs being much more efficient in explaining the data. The plateau observed in the log-likelihood curve for the CN model indicates the existence of a range of correlations that mostly represent redundant parameters.

Figure 5b shows the training, ${\mathrm{P}}({\varvec{\mathscr{D}}}_{{\mathrm{t}}}|{\mathrm{PGM}}_{{\mathrm{t}}})$, and validation, ${\mathrm{P}}({\varvec{\mathscr{D}}}_{{\mathrm{v}}}|{\mathrm{PGM}}_{{\mathrm{t}}})$, log-likelihoods for CNs and BNs. As one can see in the plot, the log-likelihood of ${\mathrm{P}}({\varvec{\mathscr{D}}}_{{\mathrm{t}}}|{\mathrm{PGM}}_{{\mathrm{t}}})$ in Fig. 5b is consistent with that of ${\mathrm{P}}({{\varvec{\mathscr{D}}}}_{{\mathrm{c}}}|{\mathrm{PGM}}_{{\mathrm{c}}})$ (in Fig. 5a) for both BN and CN, showing that the PGMs learnt from the (halved) training sets ${{\varvec{\mathscr{D}}}}_{{\mathrm{t}}}$ are as good as those obtained with the complete set of data, ${\varvec{\mathscr{D}}}_{{\mathrm{c}}}$, in both type of networks. As for the validation, the log-likelihood of ${\mathrm{P}}({{\varvec{\mathscr{D}}}}_{{\mathrm{v}}}|{\mathrm{PGM}}_{{\mathrm{t}}})$ shows that both CNs and BNs exhibit an ‘optimal’ size for which the excluded validation data is explained best. PGMs with a number of edges (parameters) above the optimum are overfitting the data that were used to train the models and fail to generalize out-of-sample (validation) datasets. The log-likelihood curve of the BN model declines after the maximum, located around 1,795 edges. Indicating that the PGM is performing worse as we include more edges. Similarly, the log-likelihood curve of the CNs declines after a maximum at 3,118 edges. Note that, for CNs, in the range of network sizes where Fig. 5a showed a plateau, the validation log-likelihood declines dramatically. Therefore, CNs with a correlation threshold above $\tau = 0.56$ result in a generalizable PGM. Edges/parameters for $\tau$ between 0.20 and 0.56 still represent relatively strong correlations but these CNs are not generalizable to explain new data. The test log-likelihood curve (inset of Fig. 5b) has a small revival when edges with correlation smaller than $\tau = 0.2$ are added. One may conclude that, in CNs, relatively strong correlations are not always relevant and small correlations are not always negligible when constructing the corresponding PGM. This is due to the mixing of strong but short and weak but relevant long-range spatial correlations, which significance CNs cannot capture effectively. Placing links/parameters by a CNs approach easily leads to overfitting of high correlations and underestimation of the effect of small (but physically important, teleconnections in this case) dependencies.

### Estimating conditional probabilities

The estimation of conditional probabilities (as a part of probabilistic analysis, box 4 in Fig. 1) is one of the key problems in machine learning and a number of methodologies have been proposed for this task, such as regression trees^37^ or Support Vector Machines^38^. Multivariate Gaussian distributions provide a straightforward approach to this problem allowing to estimate the impact of an evidential variable $X_{\mathrm{e}}$ (with known value) to other variables (gridboxes in this study). For example, assuming warming conditions in a particular gridbox of the globe $X_{\mathrm{e}}$ (e.g. a strong increase in temperature, say $X_{\mathrm{e}} = 2\sigma _{X_{\mathrm{e}}}$) the conditional probability of the other gridboxes ${\mathrm{P}}(X_i|X_{\mathrm{e}})$ provides a quantification of physical impact of this evidence in nearby or distant regions. This will allow, for instance, to study teleconnections of $X_{\mathrm{e}}$ with other distant regions.

**Figure 6:**
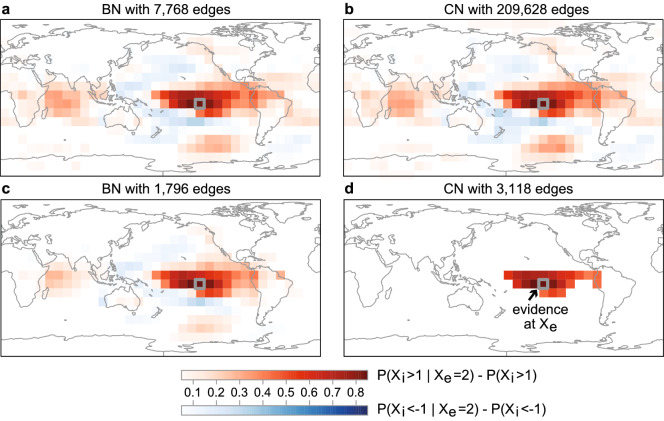
Differences of conditional and marginal probabilities ${\mathrm {P}}(X_i \ge 1{\mathrm {|}}X_{\mathrm{e}} = 2) - {\mathrm {P}}(X_i \ge 1)$ (red scale) and ${\mathrm {P}}(X_i \le 1{\mathrm {|}}X_{\mathrm{e}} = 2) - {\mathrm {P}}(X_i \le 1)$ (blue scale) as modelled with Bayesian PGMs with (**a**) 7,768 and (**c**) 1,796 edges and correlation PGMs with (**b**) 209,628 and (**d**) 3,118 edges. The location of the evidence variable $X_{\mathrm{e}}$ is denoted with a grey box and labelled in (**d**) with “Evidence at $X_{\mathrm{e}}$”. The event $X_{\mathrm{e}} = 2$ indicates a positive deviation of the mean value of twice its standard deviation, i.e. strong warming in $X_{\mathrm{e}}$. The Bayesian (**a**) and Correlation PGM (**b**) in the first row are heavily parametrized. The Bayesian (**c**) and Correlation PGM (**d**) in the second row correspond to cross-validated optimal PGMs. Maps in this Figure were created using the R-package visualizer v1.5.1 that forms part of the climate4r open framework (http://www.meteo.unican.es/climate4r)^29^.

We illustrate the performance of correlation and Bayesian PGMs to estimate conditional probabilities with the case study of the east Pacific ocean teleconnections—El Niño—Southern Oscillation teleconnections^39^—, selecting a particular gridpoint $X_{\mathrm{e}}$ in the equatorial pacific (grey box in Fig. 6a–d). A single map in Fig. 6 visualizes the conditional probabilities of warming and cooling conditions for all other gridboxes $X_i$ (i.e., ${\mathrm{P}}(X_i \ge \sigma _{X_i}|X_{\mathrm{e}} = 2\sigma _{X_{\mathrm{e}}})$ and ${\mathrm{P}}(X_i \le \sigma _{X_i}|X_{\mathrm{e}} = 2\sigma _{X_{\mathrm{e}}})$, respectively) given very warm conditions at $X_{\mathrm{e}}$. The four different maps display the results of four PGMs, corresponding to two BNs and two CNs. In Figure 6a, b the results for heavily parametrized (heavily overfitted) Bayesian (7,768 edges) and correlation (209,628 edges) PGMs are shown. These models exhibit similar results, showing warm deviation in teleconnected regions in the Indic and Southern Pacific and Atlantic oceans. The existence of these teleconnections is in agreement with the literature^39^. Figure 6c, d show the probabilities for the networks with optimum size according to our cross-validation tests (i.e., Fig. 6c a BN with 1,796 edges and Fig. 6d a CN with 3,118 edges). The correlation PGM in panel Fig. 6d only captures local deviations (note the low value of ${\mathrm{P}}({\varvec{\mathscr{D}}}_{\mathrm{c}}|\mathrm{PGM}_{\mathrm{c}})$ in Fig. 5a). Teleconnections are only quantitatively captured in association with CNs of greater size with higher log-likelihood ${\mathrm{P}}({\varvec{\mathscr{D}}}_{\mathrm{c}}|\mathrm{PGM}_{\mathrm{c}})$, however, as shown in the above section, these models are highly overfitted and, therefore, lack of generalization capabilities (i.e. they can only explain the data used for training and do not posses generalizable physical relationships—generalizable teleconnections in this case—). Drawing conclusions on the strength of the captured (teleconnected) dependencies and making decisions on the basis of predictions in CNs of large sizes is therefore questionable, if not plain wrong, as shown by our cross-validation tests above. On the other hand, the cross-validated optimum BN with 1,796 edges in Fig. 6c does capture the teleconnections (with smaller probability in some cases) and ${\mathrm{P}}({\varvec{\mathscr{D}}}_{\mathrm{v}}|{\mathrm{PGM}}_{{\mathrm{t}}})$ gives higher values as compared with those in the CNs, therefore, it is generalizable to explain new data. The reason why probabilities are a bit smaller for some teleconnected regions (with respect to the heavily parametrized model in Fig. 6a) is the non-stationary nature of El Niño events, which can take various forms, e.g., the Cold Tongue Niño event  and the Warm Pool (Modoki^40^) Niño event^41^, the former exhibiting stronger surface temperature teleconnection with the Indian Dipole and the latter with the teleconnected regions at higher latitudes^41–45^. Low but non-zero probability on significant deviation in teleconnected regions is, thus, a truthful presentation of the impact of the evidence if this is to be generalized to different El Niño types co-existing in the dataset.

## Discussion

Networks are the main subject of study in complex network theory, whereas from a machine learning perspective networks has been supporting tools to obtain probabilistic models. In this work we show that BNs, developed by the machine learning community, constitute an extremely appealing and sound approach to build complex data-driven networks based on a probabilistic framework. The BN approach provides an optimal, non-redundant, probabilistic Gaussian model of the complex system of interest using a network support characterizing the relevant dependencies. The resulting networks are sparse but rich in topological information as shown by standard complex network measures, while the probabilistic counterparts are sound models generalizable to new data and, therefore, have predictive power. In contrast, we have shown that the most common approach to graphically model complex systems, based on CNs constructed from pairwise correlations, is prone to overfitting, depends on an arbitrary threshold, and performs very poorly when one intends to generalize to explain new data, therefore, lacking of predictive power. We have shown that sparse networks that have predictive power are particularly useful when studying long distant connections in a complex system. We studied the teleconnections that occur in a complex climate dataset and showed that BNs without further post-processing faithfully reveal the various long-range teleconnections relevant in the dataset, in particular those emerging in El Niño periods.

We proposed to find the optimal size (number of edges) of a network simply and generically from the corresponding log-likelihood of the data-driven PGM. The log-likelihood measures the ability of a PGM to explain the data. Log-likelihood plots clearly show that there exists a region where the gain in explanatory power by adding more parameters/edges dramatically slows down, as reflected by a change in the slope of the log-likelihood curve in Fig. 5. A probabilistic model, either BN or CN based, begins to be overfitted once the log-likelihood curve bends, giving an objective, non biased, estimate for the optimal number of edges that need to be used. For both BNs and CNs, it turns out that including more edges results in little gain in log-likelihood and tends to produce progressively overfitted models, leading to less capability for explaining new data. We have shown that CNs need to go well above this optimum in order to capture weak, but important, teleconnections; while BNs capture the significant (even if small) teleconnections early on, when only a few hundreds of edges have been included in the model.

In addition, we uncovered that the edge-betweenness community structure of BNs attains nearly maximal entropy, $S_{{\mathrm{max}}} = \log _2 N_c$, when the number of edges is around the optimum, no matter the number of communities $N_c$. In this sense, the optimum number of parameters (number of edges) in a BN is an objective non arbitrary quantity. From an information theory perspective, this means that each assignation of a node to a given community gives maximal information about the community structure, reflecting the fact that virtually no edge is redundant. In contrast, the entropy of the community partition for CNs is far below the maximum, unless several tens (or even hundreds, depending on the correlation threshold) of communities are taken into account.

The choice of the threshold, i.e. determining the amount of edges, in CNs is problematic. Donges et al.^16,17^ already noted that to capture, for example, teleconnections with topological measures the correlation threshold has to be chosen below some maximum value. This small threshold does not coincide with that needed for the network to be statistically most significant. In^16^ various thresholds are chosen in function of topological measures to ensure a balance between the structural richness that is unveiled by the measure and the statistical significance of the network. Usually, once the threshold is chosen, this choice is justified by conducting a robustness analysis testing the effect of the threshold on the qualitative results and/or assuring a minimum level of significance—using significance tests based on randomly shuffled time series, Fourier surrogates and twin surrogates^16,17,25^. This approach poses several problems for the practical construction and interpretation of these models. Recent studies, mostly in the context of extreme rainfall data, thus propose to include an extra *ad-hoc* post-processing step in the network construction phase, in which insignificant edges (probable to occur in a random network) are removed from the final network in order to alleviate the problems introduced by redundancy^18–20,46^; Boers et al.^20^ even correct their extreme rainfall network data two times, firstly, by keeping only significant links with respect to a random network and later removing links that are not part of a ‘link bundle’, i.e., not ‘confirmed by other links’. As shown by the present work, there is a fundamental difference between the construction of CNs and that of BNs. On the one hand, CNs capture ‘strong relationships’ early on in the construction process and are affected by troublesome overfitting problems that would eventually need to be ‘cured’ by some some type of post-processing to maintain only the statistical significant ones among them—a job for which no general unbiased solution exists. On the other hand, the BN construction we propose here only captures statistically significant relationships (no matter if weak or strong) and reveals which of them are essential for increasing the explanatory capability of the model (evidence propagation).

To avoid erroneous generalization of the relative strength of significant connections in complex networks we advocate in this paper for the use of BNs, which generically yield sparse, non redundant, maximal information containing, and generalizable networks suitable for extracting qualitative information with complex measures, but that also explain new data and do not require any *ad-hoc* extra correction steps.

## Methods

### Determining CN structure from data

A CN is built with the help of the sample correlation matrix $\varvec{Q}$, which can be calculated from the anomaly data sample as follows. Let $\{d_{i}^k\}$, $\{d_{j}^k\}$, $k \in \{1,\ldots ,n\}$ be samples of size *n*—in our case $n=12\times 30=360$—of the components $X_i$ and $X_{j}$ of the random multivariate variable ${\mathbf {X}}$. The sample correlation coefficient between $X_i$ and $X_j$ is given by:

$$Q_{ij} = \frac{\sum _{k=1}^{n} d_{i}^k \, d_{j}^k}{\sqrt{\sum _{k=1}^{n}({d_{i}^{k}})^2}\sqrt{\sum _{k=1}^{n}({d_{j}^{k}})^2}} ,$$

where we have used that $\sum _{k} d_{i}^{k} =0$ for anomalies. A threshold $\tau$ is set on $\varvec{Q}$. Correlations below $\tau$ are considered as too weak and/or insignificant (these two concepts are not to be confused) to be displayed in the network. With this threshold one defines the network constrained sample matrix $\varvec{Q}_\tau = \varvec{Q}(|Q_{ij}|\ge \tau )$ in which all entries ${Q_{\tau }}_{ij}$ such that $|Q_{ij}| < \tau$ are set to zero. The adjacency matrix $\varvec{A}$ of an unweighted CN graph is then deduced by converting $\varvec{Q}_\tau$ to binary format, i.e. $A_{ij} = 1$ if ${Q_{\tau }}_{ij} \ne 0$ and $A_{ij} = 0$ if ${Q_{\tau }}_{ij} = 0$. Networks of different size (number of edges) are constructed by varying $\tau$. In this work we considered 100 CNs, obtained by varying $\tau$ in the range from 0 to 0.99.

### Learning BN structure from data

A BN is estimated with the help of a structure learning algorithm that finds the conditional dependencies between the variables and encodes this information in a DAG. Graphical (dis-)connection in the DAG implies conditional (in-)dependence in probability. From the structure of a BN a factorization of the underlying joint probability function ${\mathrm{P}}(\mathbf {X})$ of the multivariate random variable ${\mathbf {X}}$ (as given by Eq. (4)) can be deduced. We shall come back to this factorization in "Probabilistic BN models" section where we explain how networks can be extended to their corresponding Probabilistic Graphical Models (PGMs).

In general there are three types of structure learning algorithms: constrained-based, score-based, and hybrid structure learning algorithms—the latter being a combination of the first two algorithms.

Constrained-based algorithms use conditional independence tests of the form ${\mathrm{Test}}(X_i,X_j|{\mathrm{S}}_{X_i,X_j};{\varvec{\mathscr{D}}})$ with increasingly large candidate separating sets ${\mathrm{S}}_{X_i,X_j}$ to decide whether two variables $X_i$ and $X_j$ are conditionally independent. All constraint-based algorithms are based on the work of Pearl on causal graphical models^47^ and its first practical implementation was found in the Principal Components algorithm^48^. In contrast, score-based algorithms apply general machine learning optimization techniques to learn the structure of a BN. Each candidate network is assigned a network score reflecting its goodness of fit, which the algorithm then attempts to maximise^49^. Somewhere else some of us^23^ compared structure learning algorithms belonging to the three different classes on accuracy and speed for the climate dataset used here. We found that score-based algorithms perform best for the complex data in the climate data set. Algorithms in this class are able to handle high-variable-low-sample size data and find networks of all desired sizes. Constrained-based algorithms can only model complex data up to a certain size and, as a consequence, for climate data they only reveal local network topology. Hybrid algorithms perform better than constrained-based algorithms on complex data, but worse than score-based algorithms.

**Figure 7:**
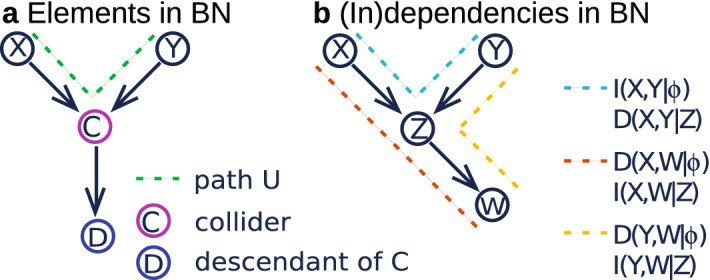
(**a**) Nomenclature of elements in a Bayesian Network (BN) and (**b**) Some (in)dependencies in a simple BN consisting of the nodes *X*, *Y*, *Z* and *W*. Two sets of nodes are dependent given a third if conditions (1) and (2) in the main text are fulfilled. In (**b**), on the one hand, the conditional relationship *X*, *Y*|*Z* and the marginal relationships $X,W|\emptyset$ and $Y,W|\emptyset$ satisfy conditions (1) and (2), so that we have D(*X*, *Y*|*Z*), ${\mathrm{D}}(X,W|\emptyset )$ and ${\mathrm{D}}(Y,W|\emptyset )$. On the other hand, the marginal relationship $X,Y|\emptyset$ violates condition (1) and the conditional relationships *X*, *W*|*Z* and *Y*, *W*|*Z* violate condition (2), so that we have ${\mathrm{I}}(X,Y|\emptyset )$ and I(*X*, *W*|*Z*) and I(*Y*, *W*|*Z*). **Proof of** $\varvec{\mathrm{D}(X,Y|Z)}$**—Conditional dependence of** ***X*** **and** ***Y*** **given** ***Z*** **in Fig.** 7**b**. The conditioning set S exists of *Z*. The only path between *X* and *Y* is the blue path. Hence we declare the blue path U. *Z* is a collider and *Z* is in S. There are no other colliders on U. Hence condition (1) is satisfied. *Z* is the only variable on U. And *Z* is a collider. Thus, U does not contain non-colliders. Hence condition (2) is satisfied. As condition (1) and (2) are satisfied we have that *X* and *Y* are dependent given *Z*, i.e. D(*X*, *Y*|*Z*).

In the following we describe how a DAG, found by a structure learning algorithm, encodes conditional dependencies. New nomenclature is indicated with an asterisk and illustrated in Fig. 7a. Two nodes *X* and *Y* are conditionally dependent given a set S (denoted by D(*X*, *Y*|S)) if and only if they are graphically connected, that is, if and only if there exists a path $\text {U}^*$ between *X* and *Y* satisfying the following two conditions:

- *Condition (1)* For every $\hbox {collider}^*$ C (node C such that the part of U that goes over C has the form of a V-structure, i.e., $\rightarrow \mathrm{C} \leftarrow$) on U, either C or a $\hbox {descendant}^*$ of C is in S.
- *Condition (2)* No non-collider on U is in S.

If the above conditions do not hold we call *X* and *Y* conditionally independent given the set S (denoted by I(*X*, *Y*|S)). Marginal dependency between two nodes can be encoded by any path U with no V-structures. In Fig. 7b six conditional (in)dependence statements are highlighted in a simple DAG. In the caption of Fig. 7 one of the statements is proved at the hand of conditions (1) and (2).

In this work we use a simple score-based algorithm, the Hill Climbing (HC) algorithm^49^, to learn BN structure. The HC algorithm starts with an empty graph and iteratively adds, removes or reverses an edge maximizing the score function. We used the Bayesian Information Criteria (BIC) (corresponding to ${\mathrm{BIC}}_0$ in^23^) score, which is defined as:

1 $$\begin{aligned} {\mathrm{BIC}}({\mathscr{G}}; {\varvec{\mathscr{D}}}) = \sum _{i=1}^N \left[ \; \log {\text {P}}(X_i {\text {|}}\Pi _{X_i}) - \frac{|\Theta _{X_i}|}{2}\log N \;\right] , \end{aligned}$$

where ${\mathscr{G}}$ refers to the graph (DAG) for which the BIC score is calculated, P refers to the probability density function that can be deduced from the graph (see "Probabilistic BN models" section), $\Pi _{X_{i}}$ refer to the parents of $X_i$ in the graph (i.e. nodes *Y* with relation $Y \rightarrow X_i$ in the graph) and $|\Theta _{X_{i}}|$ is the amount of parameters of the local density function ${\text {P}}(X_i {\text {|}}\Pi _{X_i})$. For topological analysis an undirected adjacency matrix $\varvec{A}$ can be deduced from a DAG with adjacency matrix $\varvec{A}^{*}$ with the following rule; $A_{ij} = A_{ji} = 1$ if $A^{*}_{ij} = 1$ or $A^{*}_{ji} = 1$. The size of a BN can be controlled by the number of iterations of the structure learning algorithm. In this work we obtain around 80 BNs of different sizes by saving the resulting DAGs every 100 iterations of the learning algorithm.

### Betweenness centrality

The betweenness centrality measures the extent to which a node lies on paths between other nodes^32^. A node is assigned high betweenness centrality if it is traversed by a large number of all existing shortest paths (geodesics). We define $g_{jk}$ as the total number of geodesics between node $X_j$ and node $X_k$ and $g^i_{jk}$ as the number of geodesics between node $X_j$ and node $X_k$ that include $X_i$. Then, the betweenness centrality ${BC}_i$ of node $X_i$ can be expressed as

2 $$\begin{aligned} {BC}_i = \sum _{j, k \ne i}^{N} \frac{g^i_{jk}}{g_{jk}}, \end{aligned}$$

with the convention that $g^i_{jk}/ g_{jk} = 0$ if both $g^i_{jk}$ and $g_{jk}$ are zero.

### Community detection

A community (also group or cluster) is formed by sets of nodes that are tightly knit with many edges to other nodes inside the set, while there are few edges connecting the set with other sets. A transparent way of finding communities is to look for edges that lie between communities and remove them. In this way one is left with just the isolated communities. One can detect ‘edges between communities’ noting that those edges typically have high values of edge betweenness centrality. Edge betweenness of a given edge is defined^32^ in a similar matter as the node betweenness in Eq. (2); instead of defining $g^i_{jk}$ as the number of geodesic paths that run along a node, $g^i_{jk}$ computes the number of geodesic paths that run along the edge *i* and the sum is over all nodes $j \ne k$. Based on this definition one expects that edges between communities have higher values of edge betweenness with respect to those that are not between communities because all geodesics between two nodes in two different communities go over the first. The betweenness-based-community detection algorithm is then as follows: The algorithm^50,51^ starts with one community that contains all nodes, then iteratively splits this giant community in other communities by removing edges with the highest edge-betweenness value partitioning the network in smaller communities, step by step. This continues until all nodes are singleton communities. In the process edge betweenness values of edges will change because shortest paths are rerouted after an edge removal, hence the edge betweenness values are recalculated at every step. The splitting process can be represented in a dendrogram, showing the division of larger communities into smaller ones at every stage of the algorithm evolution.

### Entropy

In order to quantitatively measure the size distribution of communities we compute the entropy^52^ of any given community partition. At a given level of the partitioning process we label the existing communities with $\alpha = 1, \dots , N_c$, where $N_c$ is the number of communities, and define the entropy of the community partition as $S = - \sum _{\alpha =1}^{N_c}\omega _\alpha \log _2\omega _\alpha$, where $\omega _\alpha$ is the fraction of nodes that belong to the $\alpha$-community. This entropy is a measure of the amount of information encoded in the community size distribution. If we were to store the complete community list and its members by specifying to which community, $\alpha = 1, \dots , N_c$, each site, $i = 1, \dots , N$, belongs to then *S* would tell us the average amount of information, $N S(N_c)$, that would be required to do the job. The entropy is maximal when the *N* network sites are evenly distributed among the available $N_c$ communities. This corresponds to $\omega _\alpha = \omega$, where $\omega = 1/N_c$ for all $\alpha$, then the entropy becomes $S_{{\mathrm{max}}} = -N_c \, \omega \log _2\omega = \log _2 N_c$. Any entropy below this number means the sizes of communities are uneven, more so the lower the entropy. A lower entropy for a community partition means less information content is stored in the community structure.

### Probabilistic Gaussian graphical models (PGGMs)

The term refers to the choice of a multivariate Gaussian joint probability density (JPD) function to associate graph edges with model parameters in a given PGM, such that the probabilistic model encodes in the JPD function a large number of random variables that interact in a complex way with each other by a graphical model. A graphical model exists from a graph and a set of parameters. The set of parameters characterize the JPD function and are reflected in the corresponding graph by nodes and edges. The multivariate Gaussian JPD function can take different forms in which dependencies between the variables are described by different types of parameters. Hence, one might build various PGGMs that could encode the multivariate Gaussian JPD function. We describe in some detail two types of PGGMs, in which parameters reflect respectively marginal dependencies and general conditional dependencies (marginal dependencies are special forms of conditional dependencies).

### Probabilistic CN models

The best-known representation of the Gaussian JPD function is in terms of marginal dependencies, i.e., dependencies of the form $X_i,X_j|\emptyset$ as present in the covariance matrix $\varvec{\Sigma }$. Let ${\mathbf {X}}$ be a *N*-dimensional multivariate Gaussian variable then its probability density function ${\text {P}}({\mathbf {X}})$ is given by:

3 $$\begin{aligned} {\text {P}}({\mathbf {X}}) = (2\pi )^{-N/2}\det (\varvec{\Sigma })^{-1/2}\exp \{-1/2({\mathbf {X}}-\varvec{\mu })^\top \varvec{\Sigma }^{-1}({\mathbf {X}}-\varvec{\mu })\}, \end{aligned}$$

where $\varvec{\mu }$ is the *N*-dimensional mean vector and $\varvec{\Sigma }$ the $N \times N$ covariance matrix.

The corresponding PGGM of the JPD function in Eq. (3) is the Probabilistic CN model, i.e., the probabilistic model that arises from a CN graph. For every CN graph, we find an estimator for $\varvec{\Sigma }$ as follows. We start from the constrained sample correlation matrix $\varvec{Q}_\tau = \varvec{Q}(|Q_{ij}| \ge \tau )$ (see "Determining CN structure from data" section). $\varvec{Q}_\tau$ cannot be used directly as an estimator for $\varvec{\Sigma }$, as $\varvec{Q}_\tau$ is, in general, not positive semi-definite. Instead, we define a new matrix $\varvec{Q}^F_\tau$ as the semi-definite positive matrix that is closest to $\varvec{Q}_\tau$ with the Frobenius norm. Specifically, the matrix $\varvec{Q}^F_\tau$ minimizes the distance to $\varvec{Q}_\tau$, $||\varvec{Q}_\tau - \varvec{Q}^F_\tau ||_F$, where the Frobenius norm is defined as $||\varvec{A}||_F = (\sum _{i, j} A_{ij}^2)^{1/2}$. The matrix $\varvec{Q}^F_\tau$ can be computed by using the Higham’s algorithm^53^, available in the R-package corpcor, for instance.

### Probabilistic BN models

Alternatively, the ${\text {P}}({\mathbf {X}})$ in Eq. (3) can be characterized with conditional dependencies of the form $X_i|{{{\mathscr{S}}}}$ with ${{{\mathscr{S}}}}\subseteq {\mathbf {X}}$. The representation of the JPD is then a product of conditional probability densities:

4 $$\begin{aligned} {\text {P}}(X_1,\dots ,X_N) = \prod _{i=1}^{N} {\text {P}}_i(X_i {\text {|}}\Pi _{X_i}) \end{aligned}$$

with

5 $$\begin{aligned} {\text {P}}(X_i {\text {|}}\Pi _{X_i}) \sim {{{\mathscr{N}}}}\left( \mu _i + \sum _{j|X_j \in \Pi _{X_i}}\beta _{ij}(X_j-\mu _j), \; \nu _i\right) \end{aligned}$$

whenever the set of random variables $\{X_i {\text {|}}\Pi _{X_i}\}_{i\in N}$ is independent^54^. In this representation ${{{\mathscr{N}}}}$ is the normal distribution, $\mu _i$ is the unconditional mean of $X_i$, $\nu _i$ is the conditional variance of $X_i$ given the set $\Pi _{X_i}$ and $\beta _{ij}$ is the regression coefficient of $X_j$, when $X_i$ is regressed on $\Pi _{X_i}$. We call $\Pi _{X_i}$ the parentset of variable $X_i$.

The corresponding PGGM in this case is the Probabilistic BN model. The graph of a BN model is a $\mathrm{DAG}$ encoding the corresponding probability distribution as in Eq. (4). Each node corresponds to a variable $X_i \in {\mathbf {X}}$, the presence of an arc $X_j \rightarrow X_i$ implies the presence of the factor ${\text {P}}_i(X_i|\dots X_j \dots )$ in ${\text {P}}({\mathbf {X}})$, and thus conditional dependence of $X_i$ and $X_j$. Moreover, the absence of an arc between $X_i$ and $X_j$ in the graph implies the absence of the factors ${\text {P}}_i(X_i|\dots X_j \dots )$ or ${\text {P}}_j(X_j|\dots X_i \dots )$ in ${\text {P}}({\mathbf {X}})$ and, thus, the existence of a set of variables ${{{\mathscr{S}}}} \subseteq {\mathbf {X}}\backslash \{X_i,X_j\}$ that makes $X_i$ and $X_j$ conditionally independent in probability^11,36^.

The structure of the BN identifies the parentset $\Pi _{X_i}$ in Eq. (4). With this structure available, one easily learns the corresponding parameter set $(\varvec{\beta} ,\varvec{\nu} )$; in our case parameters $\beta _{ij}$ and $\nu _i$ are a maximum likelihood fit of the linear regression of $X_i$ on its parentset $\Pi _{X_i}$. We use the appropriate function in the R-package bnlearn^55^. The challenge of learning the graph structure is explained in "Learning BNs structure from data" section.

### Log-likelihood definition and calculation

The likelihood of the data ${\varvec{\mathscr{D}}}$, given a model ${\mathscr{M}}$ is the density of the data under the given model ${\mathscr{M}}$: ${\mathrm {P}}({\varvec{\mathscr{D}}}{\mathrm {|}}{\mathscr{M}})$. For discrete density functions the likelihood of the data equals the probability of the data under the model. The likelihood is almost always simplified by taking the natural logarithm; continuous likelihood values are typically small and differentiation of the likelihood function (with the purpose of a maximum likelihood search) is often hard. Log-likelihood values can be interpreted equally when the expression is used for model comparison or maximum likelihood search as the natural logarithm is a monotonically increasing function.

In the following we explain the calculation of the log-likelihood ${\mathscr{L}}({\varvec{\mathscr{D}}}|{\mathscr{M}}) = \log {\mathrm{P}}({\varvec{\mathscr{D}}}|{\mathscr{M}})$ for a PGM (${\mathscr{M}}= {\mathrm{PGM}}$) for a dataset $\varvec{{\mathscr{D}}}$ formed by *n* independent data realizations $\varvec{{\mathscr{D}}}_k$, $k \in \{1, \dots , n\}$, of the *N*-dimensional random vector ${\mathbf {X}}$, with $\varvec{{\mathscr{D}}}_k = \{d^k_1\dots d^k_N\}$ and $d^k_i$ the *k*th realization of variable $X_i \in {\mathbf {X}}$. We have

6 $$\begin{aligned} \log {\mathrm{P}}({\varvec{\mathscr{D}}}{\text {|}}{\mathrm{PGM}})&= \log {\mathrm{P}}({\varvec{\mathscr{D}}}_1, \dots , \varvec{{\mathscr{D}}}_n{\text {|}}{\mathrm{PGM}}) = \log \prod _{k=1}^{n}{\mathrm{P}}(\varvec{{\mathscr{D}}}_k{\text {|}}{\mathrm{PGM}}) \nonumber \\&= \sum _{k=1}^{n}\log {\mathrm {P}}(\varvec{{\mathscr{D}}}_k{\mathrm {|}}{\mathrm{PGM}}) = \sum _{k=1}^{n}\log {\mathrm {P}}_{{\mathrm{PGM}}}(\varvec{{\mathscr{D}}}_k) \end{aligned}$$

with $\text{P}_{{\mathrm{PGM}}}$ the probability density function as modelled by the corresponding PGM with a Gaussian multivariate probability. In this work we considered two types of PGMs, correlation and Bayesian PGMs, deduced from CNs and BNs graphs, respectively. In the case of correlation PGMs, from Eq. (3), we get:

7 $$\begin{aligned} {\mathscr{L}}_{{\mathrm{CN}}}({\varvec{\mathscr{D}}}{\text {|}}{\mathrm{PGM}}_{{\mathrm{CN}}})&= \sum _{k=1}^{n}\log {\mathrm{P}}(\varvec{{\mathscr{D}}}_k{\mathrm{|}}{\mathrm{PGM}}_{{\mathrm{CN}}}) \nonumber \\&= \sum _{k=1}^{n}\log \{(2\pi )^{-N/2} \det (\varvec{Q}^F_\tau )^{-1/2} \exp [-1/2(\varvec{{\mathscr{D}}}_k-\varvec{\mu })^\top (\varvec{Q}^F_\tau )^{-1}(\varvec{{\mathscr{D}}}_k-\varvec{\mu })]\}. \end{aligned}$$

Entries in the sum (7) are evaluations of the multivariate normal density function and executed with the R-package mvtnorm^56^.

In the case of a PGGM given by a BN, from Eq. (4), we have

8 $$\begin{aligned} {\mathscr{L}}_{\mathrm{BN}}({\varvec{\mathscr{D}}}{\text {|}}{\mathrm{PGM}_{\mathrm{BN}}})&= \sum _{k=1}^{n}\log {\mathrm{P}}({\varvec{\mathscr{D}}}_k{\text {|}}{\mathrm{PGM}_{\mathrm{BN}})} \nonumber \\&= \sum _{k=1}^{n}\log \prod _{i=1}^{N} {\mathrm{P}}_i(X_i = d^k_i {\mathrm{|}}\Pi _{X_i} = d^k_{\Pi _{X_i}}) \nonumber \\&= \sum _{k=1}^{n}\sum _{i=1}^{N} \log {\mathrm{P}}_i(X_i = d^k_i {\text {|}}\Pi _{X_i} = d^k_{\Pi _{X_i}}), \end{aligned}$$

where $d^k_{\Pi _{X_i}}$ is a subset of $\varvec{{\mathscr{D}}}_k$ containing the *k*th data realization of the parentset $\Pi _{X_i}$ of $X_i$. From Eq. (5) we know that the conditional univariate densities in the sum in Eq. (8) are univariate normal and we execute them with the basic R-package stats.

## Supplementary information

Supplementary material 1
