# Supplementary material for: The probabilistic backbone of data-driven complex networks: an example in climate
Source: Sci Rep. 2020 Jul 13;10:11484. doi: 10.1038/s41598-020-67970-y (PMC7359351; doi:10.1038/s41598-020-67970-y)
Supplement: Supplementary file 1 — Supplementary material 1 [file 41598_2020_67970_MOESM1_ESM.pdf]

The Probabilistic Backbone of Data-Driven Complex Networks: An example in Climate: Supplementary Figures

Catharina E. Graafland[1,*], José M. Gutiérrez[1], Juan M. López[1], Diego Pazó[1], and Miguel A. Rodríguez[1]

[1]Instituto de Física de Cantabria, CSIC–Universidad de Cantabria, Avenida de Los Castros, E-39005 Santander, Spain
[*]catharina.graafland@unican.es

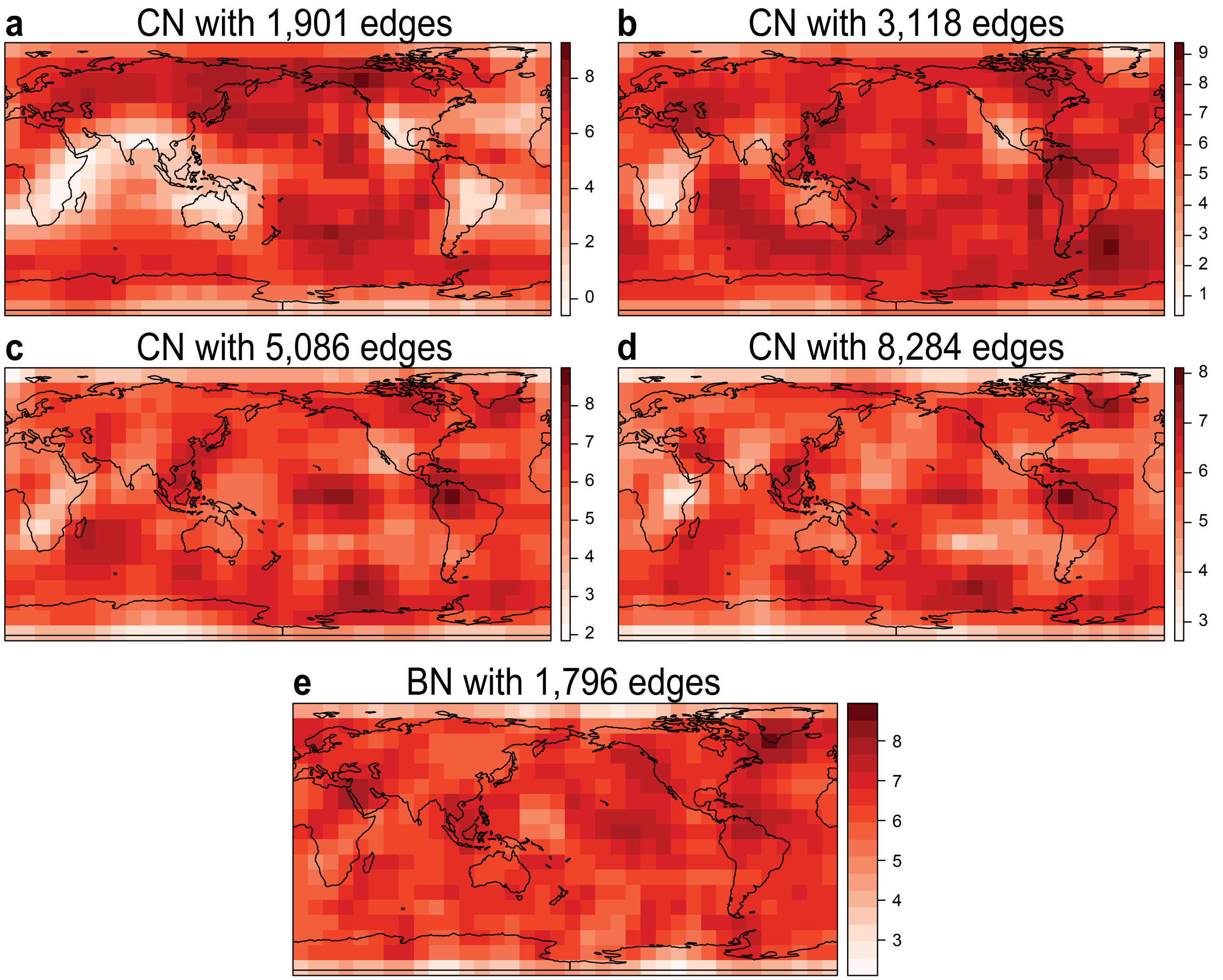

Figure 1. Maps for Correlation Networks (CNs) of size (**a**) 1,901, (**b**) 3,118, (**c**) 5,086 and (**d**) 8,284 and (**e**) Bayesian Network of size 1,796 in which gridboxes are coloured in function of their betweenness values. Raw betweenness values BC_i are transformed to $\ln(1+BC_i)$ and every gridbox presents the mean of the betweenness values of its direct neighbors for visualization purposes. CN maps (**a**)-(**d**) show alternation in the assignation of regions with high and low betweenness. The alternation between the CN maps of size (**c**) 5,086 and (**d**) 8,284 is small and one deducts a more ore less established pattern. The white boxes (zero betweenness) in CNs of size (**a**) 1,901 and (**b**) 3,118 mostly indicate variables that are unconnected to the network (see largest connected component size in Supplementary Figures 2 and 3). The white boxes in other maps indicate variables that do not belong to any geodesic. CN maps (**c**) and (**d**) share high betweenness regions in the mid-east Pacific Ocean, the Northern part of South America together with the Caribbeans, the South-West part of the Indian Ocean, the Philippines and the Chinese Sea and part of the North Atlantic Ocean against Greenland. They also share low betweenness regions for the mid-west Pacific Ocean, the west Pacific Ocean against Mexico, the Eastern part of the Indian Ocean against Australia and South-East part of the Pacific Ocean on the height of Chili. The BN map in (**e**) coincides on the above mentioned high betweenness regions. The pattern of the BN is little more flattened due to a lower value of the clustering coefficient as displayed in Figure 3. Maps in this Figure were created using the R-package VISUALIZER v1.5.1 that forms part of the CLIMATE4R open framework (`http://www.meteo.unican.es/climate4R`).

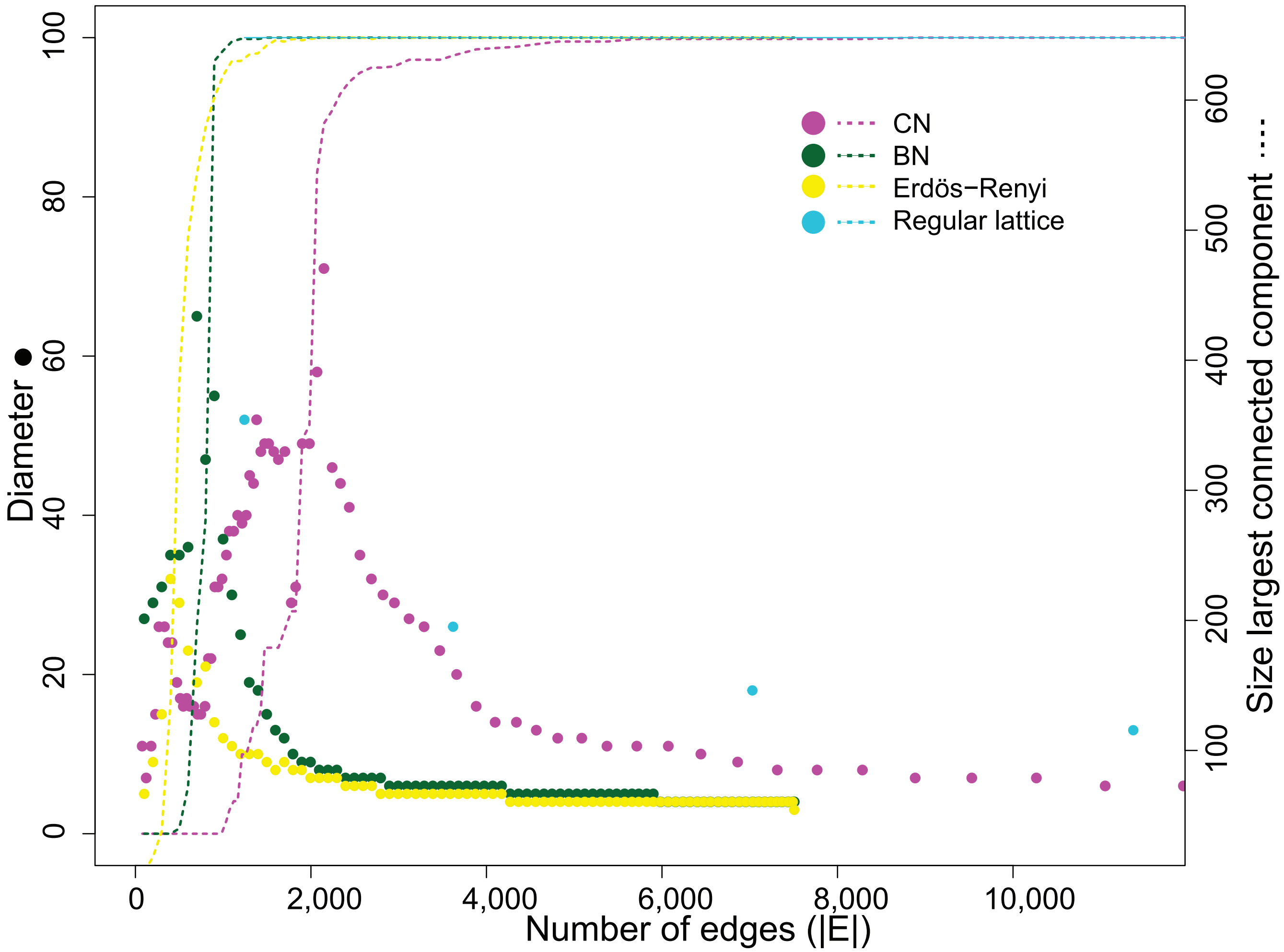

Figure 2. The diameter of a network is the length of the longest geodesic (shortest path) in the network. For a graph that is not fully connected we define the diameter as the length of the longest geodesic of the Largest Connected Component (LCC). The figure displays the diameter (left y-axis, dots) and the size of the largest connected component (right y-axis, dashed lines) versus the number of edges in Correlation Networks (CNs; magenta), Bayesian Networks (BNs; green), Erdös-Renyi random graphs (ERs; yellow) and Regular lattices (RLs; blue). ERs are random graphs in which every arc is included with probability $2|E|/(N(N-1))$ with N the total number of variables ($N = 648$) and $|E|$ the number of edges in the graph. RLs are deterministic graphs and augment locally. The smallest RL corresponds to a network in which all variables are connected with their direct neighbours in a 36×18 rectangular grid (order 1 connection). The second RL corresponds to a network in which nodes are connected with direct neighbours and neighbours of direct neighbours (order 2 connection). And so on.
A network is fully connected if the size of the LCC equals $N = 648$. All RLs are fully connected by construction. BNs, ERs and CNs are fully connected at sizes $|E| =$ 1,000, 1,900 and 5,750, respectively.
The maximum diameter value for ERs is 33 at size 500, is 65 for BNs at size 500 and is 70 for CNs at size 2,200. All maximum diameter values are found when the network is almost fully connected. BNs and CNs have similar maxima, but as BNs connect earlier, the size of the BN that yields the maximum diameter is four times smaller than that of the CN. BN diameter values first tend towards those of a local lattice (at size 500), but then tend to efficient ER values (around size 2,000). CN diameter values increase much slower during the network construction process. Values are similar to local lattice structure up to 3,500 edges and do not approach the efficient ER structure up to networks of 8,000 edges and above.

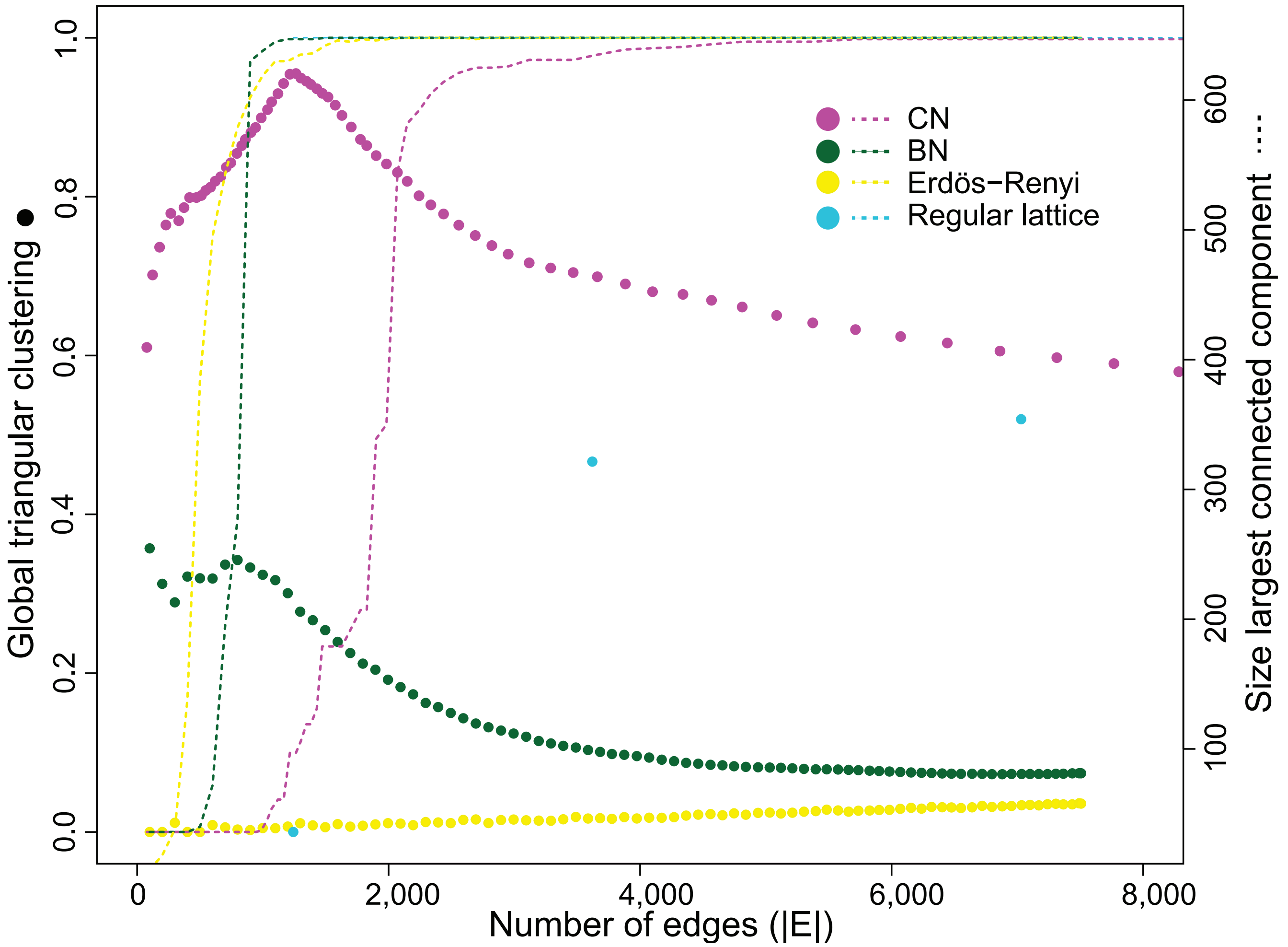

Figure 3. The global triangular clustering of a network is the ratio between the number of triangles and the total number of possible triangles that could exist: clustering = number of closed paths of length two ÷ number of paths of length two. The figure displays the clustering (left y-axis, dots) and size of the Largest Connected Component (LCC, right y-axis, dashed lines) versus number of edges in Correlation Networks (CNs; magenta), Bayesian Networks (BNs; green), Erdös-Renyi random graphs (ERs; yellow) and Regular lattices (RLs; blue). ERs are random graphs in which every arc is included with probability $2|E|/(N(N-1))$ with N the total number of variables ($N = 648$) and $|E|$ the number of edges in the graph. RLs are deterministic graphs and augment locally. The smallest RL corresponds to a network in which all variables are connected with their direct neighbors (rectangular grid; order 1 connection). The second RL corresponds to a network in which nodes are connected with direct neighbors and neighbors of direct neighbors (order 2 connection). And so on.
ERs do not possess intrinsic clustering structure, and the observed minimal grow of clustering values is only due to network saturation. RLs do have a local clustering structure. This contribution to the clustering coefficients is totally local by definition. Clustering coefficients grow in function of size. CNs of all sizes posses high clustering values. A peak value of almost 1 is obtained around 1,700 edges, even in the correlation range with relatively more large distance links clustering values remain high; clustering in CNs occurs at local and global scale (see Figure 1(**b**-**c**)). BNs posses relative low clustering coefficients when compared with CNs of similar size, however the values are significantly higher then the coefficients of ERs that do not posses clustering structure at all. The peak value 0.35 (≈ one out three connected triples is triangular) belongs to a graph that contains around 500 edges. This graph is almost fully connected, but the structure is still local. BNs with more edges have lower clustering coefficients. The BN of 1,796 edges (see corresponding network in Figure 1(**c**) has a clustering coefficient of 0.2; this BN does exhibit clustering, but the long range edges do not contribute positively to the value of the clustering coefficient as the connection between two locally clustered regions is captured with a minimal amount of long distant edges, instead of an redundant edge bundel as is the case for CNs.
